# Randomised controlled trial of a livestock productive asset transfer programme to improve economic and health outcomes and reduce intimate partner violence in a postconflict setting

**DOI:** 10.1136/bmjgh-2016-000165

**Published:** 2017-02-28

**Authors:** Nancy Glass, Nancy A Perrin, Anjalee Kohli, Jacquelyn Campbell, Mitima Mpanano Remy

**Affiliations:** 1 Johns Hopkins University School of Nursing, Baltimore, Maryland, USA; 2 Center for Health Research, Kaiser Permanente Northwest, Portland, Oregon, USA; 3 Programme d'Appui aux Initiatives Economiques (PAIDEK), Bukavu, Democratic Republic of Congo

## Abstract

**Background:**

Diverse economic empowerment programmes (eg, microcredit, village-led savings and loan, cash and productive asset transfers) for the poor have demonstrated mixed results as vehicles for improved economic stability, health and women's empowerment. However, limited rigorous evaluations exist on the impact of financial and non-financial outcomes of these programmes, especially in conflict-affected areas.

**Methods:**

The team evaluated the effectiveness of an innovative livestock productive asset transfer intervention—Pigs for Peace (PFP)—on economic, health and women's empowerment outcomes with participants in households in 10 villages in Eastern Democratic Republic of Congo. Residual change analysis was used to examine the amount of change from baseline to 18 months between the intervention and delayed control groups, controlling for baseline scores.

**Findings:**

The majority of the 833 household participants were women (84%), 25 years of age or older, married, had on average 3 children and had never attended school. At 18 months postbaseline, the number of participants in the PFP households having outstanding credit/loans was 24.7% lower than households in the control group (p=0.028), and they had an 8.2% greater improvement in subjective health (p=0.026), a 57.1% greater reduction in symptoms of anxiety (p=0.020) and a 5.7% greater improvement in symptoms of post-traumatic stress disorder (p<−0.001). At 18 months postbaseline, partnered women and men reported a reduction in experience and perpetration of all forms of intimate partner violence, although not statistically significant between groups.

**Interpretation:**

The findings support scalability of a livestock productive asset transfer programme in rural and conflict-affected settings where residents have extremely limited access to financial institutions or credit programmes, health or social services and where social norms that sustain gender inequality are strong.

**Trial registration number:**

NCT02008708.

**Key questions**

**What is already known about this topic?**

- Rigorous evaluations of microcredit programmes are limited and those that do exist provide mixed evidence on effectiveness for improving economic, health and women's empowerment outcomes.
- Productive asset transfer programmes (eg, livestock transfer) with comprehensive services can increase consumption and income for participating households.
- Limited evidence exists to guide development and evaluation of microcredit or productive asset transfer programmes in conflict-affected settings.

**What are the new findings?**

- An innovative productive asset transfer programme, Pigs for Peace (PFP), increased economic stability, improved subjective health and mental health in conflict-affected villages. Partnered men and women reported a reduction in perpetration and victimisation in all forms of intimate partner violence, although not significantly different from the control group.
- PFP has the potential to contribute to the achievement of the Sustainable Development Goals, through reducing poverty, ensuring health and achieving gender equity.
- PFP demonstrates the importance of partnerships with local expertise to transition from humanitarian ‘granting’ to household ‘investing’ for development.

**Recommendations for policy**

- Pigs for Peace (PFP) is a promising programme for improving economic stability, health and women's empowerment with hard-to-reach and underserved communities.
- Collaboration with established gender and health programmes could further develop the PFP programme and advance outcomes for participating households.
- Additional research is needed on adapting the PFP programme with diverse populations to insure the acceptability and scalability.

## Introduction

The eradication of poverty, ensuring health and well-being across the lifespan and achieving gender equity by 2030 are central to the Sustainable Development Goals (SDG).^1^ Meeting these goals require engaging the poorest families—those living on $1.25 daily and often the most marginalised within their communities—to move from insecure sources of income to more sustainable income-generating activities.^1–3^ Microcredit and productive assets programmes propose that the borrower or participant (eg, individual, household or group) operate an income-generating activity, which had previously been absent or limited by a lack of capital.^4–7^ Microcredit includes small loans ranging from $50 to $1000 and productive assets transfers are often livestock granted to households or groups serving as collateral for one another.^8^ ^9^ These financial services have been promoted as supporting business development and increase in household wealth and economic well-being of the poor.^5^ ^8^ ^10^

van Rooyen *et al*^8^ conducted a systematic review of diverse microcredit studies implemented in sub-Saharan Africa and found evidence to question the positive impacts on financial outcomes for the poorest members of communities. As the investigators noted, there were only 15 rigorously evaluated programmes, those that included a comparison group, available to include in the review. Those studies judged rigorous enough to review provided mixed impacts, including a lack of benefits for the poorest in communities and not increasing household income.^8^ The review, however, did suggest that across diverse microcredit programmes, health outcomes were improved, primarily related to reduction in days missed from work due to sickness, the number of episodes of sickness, food security and nutrition.^8^ Gaining access to and control over income-generating activities may also improve mental health because of the participant's perceived ability to meet the needs of the household, including educating and feeding children.^11–13^ Further, the review provided mixed outcomes on women's empowerment.^8^ Two studies conducted in Uganda^14^ and South Africa^15^ ^16^ showed evidence that microcredit contributes to women's decision-making power in the household and businesses. For example, the IMAGE trial in South Africa found a significant improvement in intervention, women's ability to negotiate safe sexual practices and a reduction in experiences of intimate partner violence (IPV).^16^ Although significant improvements were identified, the findings are limited by the inability to separate out the impact of the credit from gender programming, on the empowerment outcome. Although there is little evidence in conflict-affected settings, a group savings and loan intervention coupled with gender equity education in Cote d'Ivoire showed promise in preventing violence in intimate relationships and changing attitudes of men and women that favour or support a husband's use of violence to control or discipline his wife.^17^ Evaluating potential harms, such as increased violence in relationships, during economic development programmes is critical, given the potential of conflict related to shared decision-making over spending additional income or perceived threats to husband's authority in the household.^4^ ^16–18^

Recent evidence from a multicountry evaluation has advanced the use of productive asset transfer for economic development with the poor.^9^ The Graduation Programme was designed by the Bangladesh Rural Advancement Committee (BRAC) to provide a comprehensive set of services, including a grant of a productive asset (eg, livestock or market-based business) to the poorest households in a defined community.^9^ The idea is to provide a ‘big push’ over a limited period to reduce extreme poverty. The investigators reported positive findings from the grant of a productive asset with comprehensive services increased consumption and income for participating households in all countries (Ethiopia, Peru, Pakistan, Ghana, Honduras and India).^9^ In addition to the productive asset, grant services include training and support for the asset, life skills coaching, regular consumption support for a defined period of time, access to savings accounts and health information or health services.^9^ These services plus regular home visits are designed to complement the households in a productive self-employment activity.^9^ Although the findings are impressive, the comprehensive nature of the programme and services is challenging to replicate in a humanitarian and conflict-affected settings, such as rural Eastern Democratic Republic of Congo (DRC), the study setting. More relevant to the DRC context is a Zambian livestock transfer programme implemented by Heifer International that included training for male and female participants. The physical transfer or ‘granting’ of livestock (cow or goats) with training on gender, nutrition, livestock health and care, sustainability, accountability resulted in increased cooperation and shared decision-making on the use of resources between men and women in participating households.^19^

DRC provides an exemplar of the ways in which prolonged conflict, human rights violations and the related negative health, economic and social consequences can impact communities.^20–24^ Violence against civilians is used as a ‘deliberate and strategic tactic in war’,^25^ to destroy or expel populations and pillage land and livestock. The rural territory targeted for the study reported the loss of land and essential tools for farming, resulting in limited agricultural productivity.^26^ ^27^ The looting or loss of animals has also limited the household's ability to pay for health needs, school fees and economic shocks (eg, death of a family member) or opportunities (eg, marriages, births).^26^ ^28^ As agricultural production and animal husbandry has decreased in rural Eastern DRC in the past two decades of conflict,^26^ ^29^ a cycle of food insecurity,^30^ poor health and extreme poverty^29^ ^31^ has likely been further aggravated by exposure to multiple traumatic events with limited access to quality financial and support services.^32^ The daily economic, health and resource constraints on rural populations in addition to the trauma of violence and forced displacement due to conflict add stress to family and social relationships^33^ and may result in conflict and violence of wives by husbands.^34^ The study authors previously reported on the potential of livestock/animal assets to moderate mental health symptoms for women multiple conflict-related traumatic events.^13^ As the household livestock/animal assets increased, the impact of conflict-related traumatic events on symptoms consistent with post-traumatic stress disorder (PTSD) and depression was reduced. Livestock/animal assets extend beyond its association with household wealth, as other measures of wealth (eg, durable housing, savings, regular work) did not buffer the effect of conflict-related traumatic events on mental health symptoms. Women reported using the funds gained through the livestock/animal asset to pay for school fees, purchase land and materials to build/repair homes, thus potentially strengthening self and household perception of productivity and status and advancing well-being.^13^

In 2010, Programme d'Appui aux Initiatives Economiques (PAIDEK), a Congolese microfinance organisation and the Johns Hopkins School of Nursing (JHSON) joined in collaboration to improve household economic stability, health and safety in rural Eastern DRC.^27^ The partners adapted their experience in microcredit, knowledge of productive asset transfer programmes, health and women's empowerment to the DRC context and developed Pigs for Peace (PFP). PFP is a hybrid programme that integrates microcredit and productive asset transfer principles. In rural DRC, animal husbandry continues to be one of the few opportunities for economic stability as livestock are assets to accumulate to rebuild household wealth and social status.^35^ Pigs are traditional productive assets, therefore, not intended for regular food consumption but rather serve as a ‘savings account’ for economic opportunities and crises.^26^ ^28^ ^29^ The purpose of this study is to rigorously evaluate the effectiveness of PFP on financial and non-financial outcomes in a rural, conflict-affected setting.

## Methods

### Study design and participants

The objective of the study was to evaluate the effectiveness of a hybrid microcredit/livestock asset transfer programme, called PFP on economic, health and IPV outcomes in 10 villages in the Walungu Territory in South Kivu Province. The study design was a randomised community trial. We hypothesised that at 18 months postbaseline, participants in PFP households would have increased economic stability (eg, livestock/animal assets, reduced credit), improved subjective health and mental health and less conflict in the household, measured by IPV, compared with delayed control households. The delayed control groups received the pig after the 18-month data collection was completed. The trial was registered with ClinicalTrials.gov NCT02008708 in December 2013.

A participatory and realistic approach was used to identify villages in the Walungu Territory (located between 40 and 80 km from the capital of South Kivu Province) for participation in PFP. This included: (1) feasibility of delivering PFP over a wide geographical area with limited infrastructure; (2) commitment to the PFP (pig as a productive asset) by traditional chiefs and administrators after detailed discussion with the implementing partner; (3) findings from village-level assessment including review of administrative data and semistructured interviews with key stakeholders that identified the extreme poverty and vulnerability of residents with limited availability of microcredit and/or social programmes; and (4) security in the area that allowed for implementation of the intervention.

### PFP livestock microfinance intervention

PFP was developed in partnership between PAIDEK, a Congolese microfinance organisation and JHSON.^27^ PFP uses pigs as productive assets because they are an important source of economic stability and social status with no cultural or religious taboos or gender-based responsibilities related to raising, breeding or selling. The partners revised the ‘granting’ of the productive asset, a female piglet aged 2–4 months to a productive asset ‘credit’ to participating households. PFP participants agreed to build a pigpen and compost pit as well as repay the ‘credit’ by transferring two piglets from the initial litter (one to repay the original asset transfer and one to pay interest) to other members of the village association. After ‘repaying’ the two piglets, the remaining piglets and the original pig is the household's to continue to raise, breed and sell as they determine; however, the PFP staff remains available for mentorship and support.^13^ ^27^ Similar to other microcredit and productive asset transfer programmes, PFP provided practical skills training to participants on managing nutrition and care of the livestock asset, biweekly home visits by trained staff, support for association meetings and basic health services by a local veterinarian technician. Given the long-term humanitarian interventions in Eastern DRC, the local implementing partner, PAIDEK, was hesitant to provide the consumption support of a regular transfer of food or cash, fearing this would imitate the humanitarian approach and limit the participants taking responsibility for the productive asset. However, after discussion, consumption support of 50 kg of palm kernel meal to households was provided when the pig had the initial litter. This supported the provision of nutrition-rich food during the ∼2 months prior to weaning the piglets, thus supporting healthy growth and the ability of participants to ‘repay’ and transfer pig assets to additional households in the village associations.

### Eligibility and randomisation

Women and men, aged 16 years and older, were eligible for the project and study if they expressed a commitment to and understanding of PFP (‘repayment’ of pig asset with two pigs), were permanent residents of participating villages and were responsible individuals in the household. Minor participants (ages 16–17 years) were eligible if they reported being married, widowed, parent or head-of-household (eg, responsible for younger siblings or ill parent). The partners decided to avoid ‘women only’ programming as men are important members of households and their active engagement and support can influence the overall success of the household and programme. Participation in the study was limited to one eligible adult (man or woman) in the household. The eligible man or woman representing the household participated in a public lottery to randomly assign households to the intervention (first to receive the pig) and delayed control group (receive transfer of offspring from the intervention group). Eligible participants placed their name in a container and names were drawn out one-by-one by a village child supervised by the study team with the first name drawn assigned to the intervention, second to control, third to intervention, until the planned 66 households were assigned to each condition. Sixty-six households per village were planned for the study. However, due to high level of interest in PFP, a second delayed control group was formed such that a minimum of 66 households in each village were randomised (intervention and delayed control group) and the remaining eligible men and women representing households in the village were placed in the second delayed control group (which also received a transfer of an offspring from intervention group).

### Data collection procedures

Baseline data collection took place after translation and back translation of the study questionnaire in French and local languages (Swahili and Mashi), pilot testing of the questionnaire on tablet computers and randomisation of study participants, but prior to training and distribution of the female pig loan to the intervention group. The PFP questionnaire was developed to measure our primary outcomes of subjective health, PTSD, anxiety and depression and secondary outcomes of economic stability and violence/abuse (physical, sexual and psychological) perpetration and victimisation using existing, validated research instruments and findings from this team's prior research.^13^ To address the logistical challenges of working in an extremely low-resource setting, the team collected baseline data in two phases of five villages each between 21 May 2012 and 8 November 2012. The follow-up interviews took place between 7 December 2013 and 4 May 2014, ∼18 months after the first loan pigs was given to a member of the intervention group. Pig loan distribution was initiated in July 2012 after the first phase of baseline data collection was completed, and continued for 7 months as loans were given when participants completed their pigpens. The delayed control groups received the pig after the 18 months data collection was completed. Male and female PFP staff were also trained as research assistants and served as supervisors for 10 male and female Congolese research assistants hired and trained for the data collection periods (6, 12 and 18 months postbaseline) of the study. Participants reported being comfortable responding to interview questions from male or female research assistants. Training focused on human participants research ethics, interview protocols and safety, including providing referrals for health and social support for participants as needed. Since interviews were conducted when participants would be earning their daily income, compensation for their time (∼60–90 min) was provided per local rates, ∼US$1.50.

### Ethics statement

The Institutional Review Board of the Johns Hopkins Medical Institute (JHMI) approved the study on 18 November 2010 (NA_00044037) and a committee of respected Congolese educators at the Universite Catholique at Bukavu (UCB) and community members reviewed and approved the study. Interviews were initiated only after receiving oral, voluntary informed consent from the participant. Oral consent was approved during ethics committee review as the majority of our participants had never attended school, so written consent was perceived as a significant challenge and potential barrier to participation. Inclusion of eligible minors (16–17 years old) was also approved during the ethics committee review. Study identification codes and names were recorded during one-on-one interviews; all data recorded through the tablet-based program were encrypted and uploaded to a password-protected and HIPAA-certified server managed by the study team. Once uploaded, data were automatically erased from the tablet-based program. Names were centrally removed and stored in a separate file on a password-protected study computer.

### Study questionnaire

#### Demographics and household wealth

Our questionnaire was developed using validated items from previous studies, including the Intervention with Microfinance for AIDS and Gender Equity (IMAGE) study team in South Africa^16^ and the WHO Domestic Violence and Health (2005) study.^36^ We collected current demographic information from the participant on his/her marital status, educational level, regular work (yes/no), perceived household wealth in comparison to other households in the village (ie, 1=worse than others, 2=same as others, 3=better than others), dwelling details (yes/no for durable housing defined as roof made of tin, walls of wood/brick) and household savings (yes/no). We also asked participants to report on other adults and children living in the household by age and gender.

#### Economic stability and livestock/animal assets

Economic stability was measured by the number of cash and non-cash loans a participant had in the 12 months prior to baseline interview and the months prior (∼6 months) to the follow-up time point. This was dichotomised into none versus one or more loans. A total livestock/animal asset score was computed for each participant based on the number and type of animals owned to establish the value of each type of animal. The team surveyed nine livestock/animal vendors in five different village markets in the study area and collected the current price to purchase the most commonly owned livestock/animal assets. The average cost in US dollars are cows $450, pigs $70, goats $50, poultry $10, rabbits $8 and guinea pigs $1. We computed a total livestock/animal asset score for each participant's household by multiplying the average market price for the livestock/animal by the number of household livestock/animals reported at the baseline and 18-month follow-up interview.^13^ Since these scores were extremely skewed, they were recoded into quintiles based on the baseline distribution and the ordinal quintile scores were used in the analysis.

#### Traumatic events, subjective health and mental health

The exposure to trauma events section of the questionnaire was adapted from the Harvard Trauma Questionnaire (HTQ), a multipart cross-culturally validated instrument that measures traumatic events and PTSD^37^ that the team had previously used in the study setting.^13^ ^38^ Exposure to trauma was analysed as a continuous variable (0–18 different traumatic events). A 16-item version of section 4 of the HTQ^39^ was used to identify symptoms consistent with PTSD in the past 7 days. Subjective health was measured with one item, rating health from poor to excellent in past 30 days. The depression and anxiety components of the Hopkins Symptom Checklist (HSCL) were used for reporting the experience of symptoms that bothered or distressed the respondent during the past 1 month.^39^ An average symptom score for PTSD, depression and anxiety was calculated. The HTQ and HSCL have been used widely in conflict-affected and humanitarian emergencies and both have strong psychometric properties for measuring of traumatic events and symptoms consistent with PTSD and depression in conflict-affected settings.^40–42^ In this sample, Cronbach's α was 0.86 for anxiety, 0.85 for depression and 0.89 for PTSD.

#### Intimate partner violence

Women were asked about psychological abuse, physical and/or sexual violence perpetrated by their male partners; men were asked about their perpetration of IPV against their female partners. The items asked about the partner included: (1) humiliating, (2) hurting, (3) insulting, (4) pushing, (5) slapping, (6) twisting arm or pulling hair, (7) punching, (8) kicking, dragging or beating, (9) choking or burning, (10) threatening or attacking with a weapon, (11) forcing to have sexual intercourse and (12) forcing to perform other sexual acts. Binary variables were created indicating any experience/perpetration of each of the following types of IPV: psychological abuse (items 1–3), physical violence (items 4–10) and sexual violence (items 11 and 12).

*Statistical power:* We used data from Roberts *et al*^43^ study of the reliability and validity of the SF-8 with a conflict-affected population in northern Uganda as the basis for our power analyses. Since we did not have an estimate of the intraclass correlation (ICC) (individuals nested within villages), we varied the ICC from 0.001 to 0.010. For a sample size of 300 per group and assuming no change from baseline to 18 months in the control group and a 10% improvement in the intervention group, a α level of 0.05, the power is 0.94, 0.89 and 0.83 for ICCs of 0.001, 0.005 and 0.010, respectively.

### Statistical analysis

For the analyses, the intervention group (N=309) was compared with the first delayed control group (N=296) and the second delayed control group (N=228). All analyses used a generalised estimating equation approach to control for the clustering of participants within villages. The intervention and control groups were compared on baseline variables using a Gaussian distribution and identity link function for continuous variables. Those with and without missing data were compared using the same approach to determine if there were baseline factors related to missingness. The analyses were based on intention to treat with all participants included in the main analyses. Multiple imputation (with 10 imputed data sets) was used to replace missing data based on recommendations of Schafer.^44^ As a sensitivity analyses, we compared the results from multiple imputations with a completers only analysis.^45^ The main GEE model was a residualised change regression to examine the difference in continuous variables in the amount of change from baseline to 18 months between the intervention and control groups, controlling for baseline scores on the outcome. Change score on the outcome was the dependent variable predicted from a dummy variable for group (intervention vs control) and baseline score on the outcome. The residualised change model was selected over ANCOVA model because the former accounts for differences in the outcome at baseline and the latter assumes differences between the groups at baseline. The analysis accounts for any differences at baseline because the differences may affect the degree of change.

### Role of the funding source

The funding source did not play a role in the design of the study, data collection, analysis, interpretation or writing of the results. The corresponding author had full access to all the data in the study and had final responsibility for the decision to submit for publication.

## Results

### Sample description

**Figure 1:**
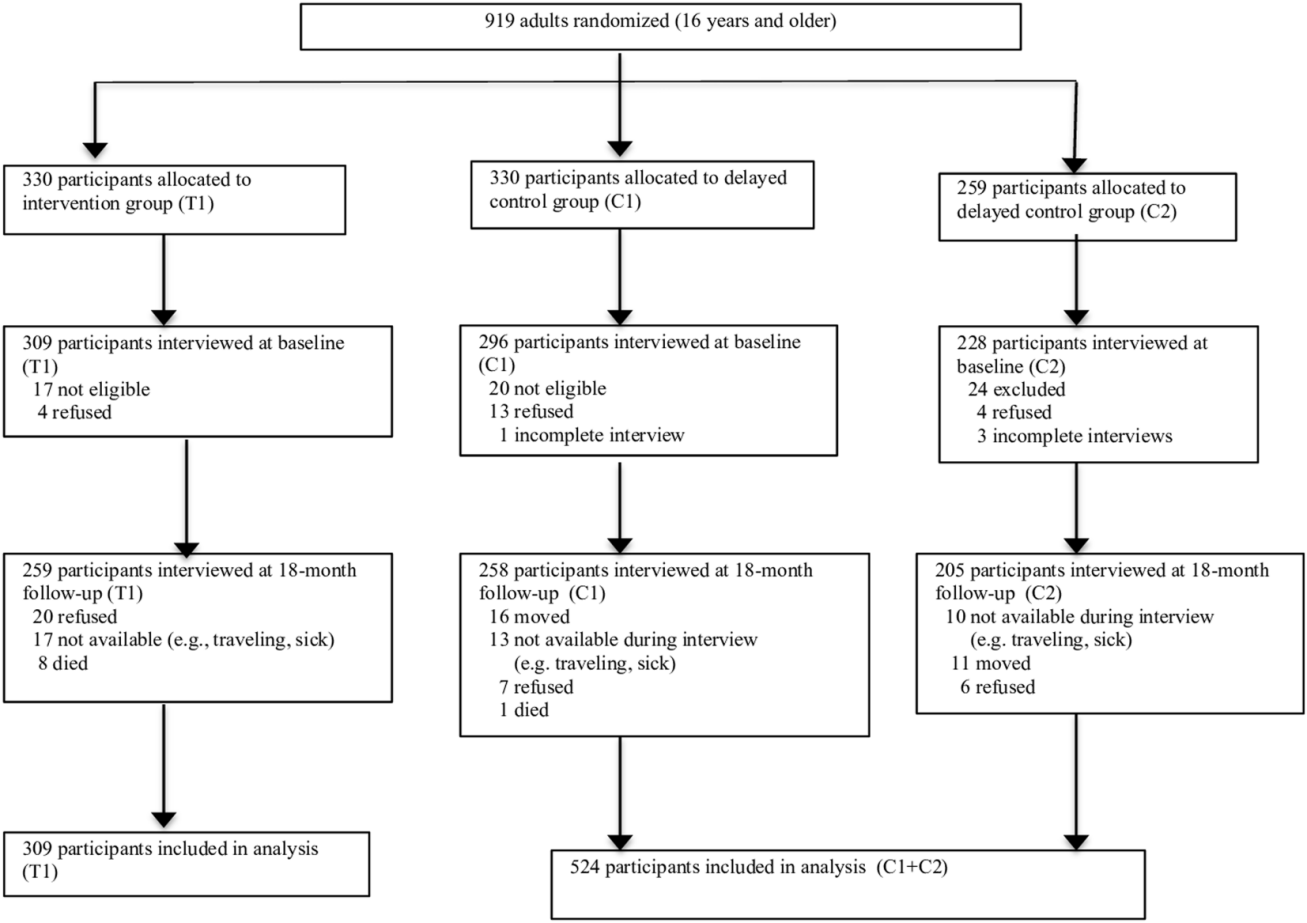
Trial profile.

The majority of the 833 household participants were women, 25 years or older, married, had on average 3–4 children in the home and had never attended school. Most participants reported living in homes with non-durable walls (N=740, 89%) and roofs (N=487, 59%). Very few (N=38, 5%) had savings; the vast majority (N=680, 82%) described their household wealth as being the same or worse off than most people in the village. The intervention and control groups were not significantly different on any of these variables at baseline (table 1). The two groups were not significantly different on any of the outcome variables at baseline, with the exception of anxiety. The control group had significantly higher anxiety than the intervention group. Lost to 18-month follow-up was not significantly different between the groups; 13.0% (N=68) of the control group and 16.2% (N=50) of the intervention group did not complete the interview (figure 1). Men (N=36, 27.7%) were more likely than women (N=82, 11.7%, p<0.00001) to not complete the 18-month interview. Completers and non-completers did not differ on age, marital status, schooling, perceived wealth, subjective health, PTSD, IPV, livestock/animal assets or having loans at baseline. Those who were lost to follow-up had significantly lower anxiety (p<0.001) and depression (p=0.015). In addition, there were no differences between the intervention and control group non-completers on the baseline characteristics.

**Table 1:** Demographics by condition

	Control group N=524	Intervention N=308	p Value
Per cent female	450 (86.0%)	251 (81.5%)	0.081
Age			0.218
15–19	9 (1.7%)	4 (1.3%)	
20–24	70 (13.4%)	37 (12.0%)	
25–34	145 (27.7%)	83 (26.9%)	
35–44	121 (23.1%)	54 (17.5%)	
45–60	146 (27.9%)	103 (33.4%)	
61+			
Marital status			0.501
Married	391 (75.0%)	224 (72.7%)	
Divorced/separated	17 (3.3%)	16 (4.2%)	
Widowed	96 (18.4%)	62 (20.1%)	
Abandoned	9 (1.7%)	2 (0.6%)	
Never married	8 (1.5%)	4 (1.3%)	
Schooling			0.312
None	339 (64.7%)	184 (59.7%)	
Did not complete primary	87 (16.6%)	49 (15.9%)	
Primary completed	86 (16.4%)	65 (21.1%)	
Secondary completed	12 (2.3%)	10 (3.2%)	
Mean number of adults living in the home (range)	2.27 (0–10)	2.41 (0–10)	0.218
Mean number of children living in the home (range)	3.38 (0–11)	3.53 (0–9)	0.530
Have a non-durable roof	308 (58.8%)	179 (58.1%)	0.852
Have non-durable walls	469 (89.5%)	271 (88.3%)	0.407
Has household savings	24 (4.6%)	14 (4.5%)	0.982
Perceived wealth worse than others	243 (46.4%)	131 (42.7%)	0.741
Subjective health* (SD)	3.79 (1.24)	3.94 (1.17)	0.079
Anxiety† (SD)	1.80 (0.55)	1.70 (0.54)	0.011
Depression† (SD)	1.82 (0.50)	1.77 (0.46)	0.149
PSTD‡ (SD)	2.19 (0.67)	2.17 (0.63)	0.632
Experienced/perpetrated psychological abuse (among those who are partnered at baseline, N=584)	161 (42.9%) (N=375)	73 (34.9%) (N=209)	0.059
Experienced/perpetrated physical violence (among those who are partnered at baseline, N=584)	72 (19.3%) (N=374)	38 (18.2%) (N=209)	0.752
Experienced/perpetrated sexual violence (among those who are partnered at baseline, N=584)	98 (26.1%) (N=376)	43 (20.7%) (N=208)	0.146
Currently have a loan	172 (32.8%)	120 (39.0%)	0.073
Animal value (median/range)	44 (0–2100)	33 (0–3710)	0.815

*Participant health measured on a 1–6 scale (1=excellent, 6=very poor).

†Measured by Hopkins Symptom Checklist (HSCL).

‡Measured by the Harvard Trauma Questionnaire (HTQ).

Table 2 summarises the baseline and 18-month outcomes by group.

**Table 2:** Baseline and 18-month means (SD), parameter estimate from the residualised change analyses and associated effect sizes

	Control (N=524)		Intervention (N=308)		Regression coefficient for group (95% CI)*	p Value	Effect size (95% CI)†
	Baseline	18 months	Baseline	18 months			
Subjective health‡	3.79 (1.24)	3.82 (1.26)	3·94 (1·16)	3.65 (1.28)	−0.188 (0.013 to 0.362)	0.035	0.15 (0.13 to 0.18)
Anxiety§	1.80 (0.55)	2.70 (0.55)	1.70 (0.54)	1.58 (0.50)	−0.086 (0.012.0.159)	0.023	0.15 (0.13 to 0.18)
Depression¶	1.82 (0.50)	1.68 (0.46)	1.77 (0.46)	1.60 (0.44)	−0.055 (−0.008 to 0.119)	0.089	0.11 (0.09 to 0.13)
PTSD**	2.19 (0.67)	1.90 (0.51)	2.17 (0.63)	1.75 (0.48)	−0.137 (0.062 to 0.211)	0.004	0.21 (0.14 to 0.23)
One or more loans	32.82%	15.86%	38.96%	9.21%	−0.575 (0.062 to 1.088)	0.028	0.13 (0.12 to 0.14)
Animal value (median)	44	10	38	70	−0.456 (0.658 to 0.254)	0.00004	0.36 (0.34 to 0.38)

*Reference group is control group.

†Effect size is based on residualised change score.

‡Participant health measured on a 1–6 scale (1=excellent, 6=very poor).

¶Measured by Hopkins Symptom Checklist (HSCL).

**Measured by the Harvard Trauma Questionnaire (HTQ).

*Economic status.* At 18 months postbaseline, the participants in PFP reported significantly greater increase in household livestock/animal assets than the control group (p=0.00004), controlling for assets at baseline. Participants in PFP were significantly less likely to have one or more loans (received as cash loan or inkind) (p=0.028) than control group participants, controlling for whether or not they had one or more loans at baseline. The same pattern of results was found with completers only. Participants reported that loans were typically received from family members or friends, health centres or small businesses in the village, only 1% of participants reported having credit with a traditional microfinance organisation.

*Physical and mental health.* PFP participants had significantly greater improvement in subjective health (p=0.035), controlling for their baseline subjective health. The intervention group also had greater improvement in symptoms of anxiety (p=0.023) and post-traumatic stress (p=0.0004), but did not differ on change in symptoms of depression (p=0.089). The same pattern of results was found with completers only.

*Intimate partner violence*. Among men and women who were partnered at baseline and 18 months (N=311 control, N=162 intervention), the groups differed on experiencing/perpetrating psychological abuse (35.1% control, 27.2% intervention; p=0.080) at 18 months although not statistically significant. Further, partnered women and men in the intervention and delayed control groups reported a decrease in experienced/perpetrated physical and sexual violence, the groups did not differ significantly on physical (p=0.340) or sexual violence (p=0.503) at 18 months. Importantly, the study was powered for the main outcomes using the entire sample (N=833); therefore, the analyses for IPV among those married at baseline and 18 months (N=473) are underpowered.

## Discussion

The study findings confirm the hypotheses that participants in PFP households would have increased economic stability and improved subjective health and mental health compared with participants in delayed control households in rural, conflict-affected villages. In rural DRC, like rural communities globally, animal husbandry continues to be one of the few opportunities for economic stability as livestock are productive assets to accumulate to rebuild household wealth and social status.^29^ ^35^ Livestock is a visible symbol of wealth, productivity and social status to the extended family and larger community. Livestock possession and productivity influences the owners' positive perception of self and household wealth.^13^ ^28^ ^29^ The local implementing partner, PAIDEK, was essential in identifying the productive asset that would result in improved economic stability and engaging men and women in the programme, as cooperation and shared decision-making was viewed as critical to success.^29^ ^46^ ^47^ For example, cows and goats were not selected as the productive asset because women cannot sell cows or goats without consent from the husband or male member of the household, as these animals are tied to the dowry system. As is tradition among the Shi people, the majority tribe in the study area, the future husband's family provides one cow to the future wives family. In recent years, with the loss of livestock wealth in rural areas, goats are often used for the dowry. Further, important in selecting the productive asset is that the vast majority of residents in the target area are either Catholic or Protestant and pork/pork products are produced and regularly consumed. Thus, the pig was the productive asset that represented a gender-neutral intervention to bring husbands and wives and other family members together in income-generating activities to improve economic stability for the household.

The findings demonstrated improved subjective health and a reduction in symptoms associated with poor mental health. Women and men in this area of Eastern DRC have experienced significant trauma over a prolonged period, resulting in symptoms of PTSD, depression and anxiety that can negatively impact productivity and family relationships.^23^ ^48–50^ PFP may have reduced these negative mental health symptoms by limiting stress through increased livestock/animals assets and less cash or inkind credit with family, friends and others.^13^ There have been other successful and innovative efforts to address unmet mental health needs through skilled healthcare. For example, Bass *et al*^48^ conducted a study with female sexual violence survivors in Eastern DRC to examine the effectiveness of an adaptation of group cognitive processing therapy (CPT) provided by community-based psychosocial assistants supervised by psychosocial staff at an international NGO and US-based clinical experts. The findings indicate that psychosocial assistants with appropriate training and supervision can implement psychotherapeutic treatments such as CPT and improve mental health for women. Our findings build on this work by demonstrating positive mental health outcomes, reduced symptoms of PTSD and anxiety for men and women participants. This is an important finding as the intervention is effective with male and female participants that had experienced multiple and diverse forms of traumatic events, beyond sexual violence. Further, it provides an example of a potentially sustainable economic programme led by village associations that has the added benefit of reducing mental health symptoms in settings that have extremely limited infrastructure and capacity to provide mental healthcare. In Eastern DRC, as in many low-resource countries, there is a lack of government-funded health centres with a workforce that has training in mental healthcare. It is estimated that DRC has 0.07 psychiatrists working in the mental health sector per 100 000 population.^51^

Although PFP does not include a women's empowerment component, our staff emphasised the importance of communication and shared decision-making between husbands and wives in the programme. Analyses of IPV are based on those who were partnered at baseline and 18 months (56.8% of the total sample) and do not have adequate statistical power; however, the pattern of reduction of IPV is clear. At 18 months postbaseline, fewer participants in the intervention group reported experiencing/perpetrating psychological abuse than the control group (27.2% intervention, 35.1% control; p=0.080). Further, reductions in physical (3.5%) and sexual (10.6%) IPV were reported by partnered women and men in the intervention group; however, the reduction was not significantly different between intervention and control group participants. Research has suggested that in conflict-affected populations, men express a need to recover their authority and role as head of household, despite the severe economic and health stress on the family.^34^ ^52^ With the DRC national prevalence of past year frequent (sometimes or often) physical, sexual and/or psychological IPV of 43.9%,^53^ identifying effective approaches to IPV prevention is critical to sustained development. Women's report of decreased IPV by their husband/partner was consistent with men's report of reduced use of IPV over the 18-month period. Indepth qualitative interviews were conducted at ∼6–9 months postbaseline with married/partnered male and female PFP participants that reported IPV perpetration or victimisation at baseline. The indepth interviews added to our understanding of risk factors associated with IPV in participating households. Men and women described financial stress including lack of work for men outside the home, alcohol use, male peer group sanctions use of IPV and social norms that support a husband's role in disciplining his wife as risk factors for husband's use of IPV.^54^ Future PFP programming will include primary prevention of IPV through engaging men and women in changing social norms that sustain gender inequality and reducing the multiple risk factors identified in our work and others that may provide additional reductions in IPV and enhance women's empowerment outcomes.

PFP prioritised the focus on transitioning from humanitarian ‘granting’ to household ‘investing’ for sustainable development. The success of PFP provides support for the importance of indigenous expertise in sustainable development programmes to improve economic stability, subjective health and mental health and reduce IPV in rural households. Our implementing partner had the expertise and access to engage traditional and administrative village leaders in productive and culturally appropriate economic activities that supported the participation of men and women.^27^ Partnerships have the benefit of also building local capacity to provide economic and other development initiatives, which is a critical step to ending a dependence on humanitarian aid and progress to sustainable development that will advance wealth, health and gender equality. It is certain that credit and productive asset transfer programmes alone will not solve the multiple challenges facing families in conflict-affected settings. However, a collaboratively developed and culturally relevant economic development programme that has the benefit of improved health and women's empowerment has potential for advancing SDG.

This study has limitations. The study was conducted in 10 conflict-affected rural villages in 1 province in Eastern DRC. Therefore, the experiences of the male and female participants are not generalisable to all rural households experiencing conflict. Participants reported exposure to multiple traumatic events within the past 10 years, representing at least two periods of conflict; therefore, some of the reported traumatic experiences were likely in the recent past and others several years prior to the baseline interview, so recall bias is an issue. Further, given the limitations of resources, our PFP programme staff was also trained as research assistants and participated in data collection and supervised interns that conducted interviews with participants across the 18 months. We also acknowledge the potential for contamination between the groups, as the delayed control group households were in the same villages as the intervention households.

## Conclusions

PFP has important implications for achieving the SDGs with positive findings that intersect areas critical to sustainable development—economic stability, improved subjective health and mental health and reduced violence against women. PFP has potential for scalability, given that it was successful in a challenging rural and conflict-affected setting where residents have extremely limited access to financial institutions or credit programmes, health or social services and where social norms that sustain gender inequality are strong.
